# Berberine Inhibition of Fibrogenesis in a Rat Model of Liver Fibrosis and in Hepatic Stellate Cells

**DOI:** 10.1155/2016/8762345

**Published:** 2016-04-30

**Authors:** Ning Wang, Qihe Xu, Hor Yue Tan, Ming Hong, Sha Li, Man-Fung Yuen, Yibin Feng

**Affiliations:** ^1^School of Chinese Medicine, Li Ka Shing Faculty of Medicine, The University of Hong Kong, Pokfulam, Hong Kong; ^2^Centre for Integrative Chinese Medicine and Department of Renal Medicine, Faculty of Life Sciences and Medicine, King's College London, London SE5 9NU, UK; ^3^Division of Gastroenterology and Hepatology, Queen Mary Hospital and Department of Medicine, Li Ka Shing Faculty of Medicine, The University of Hong Kong, Pokfulam, Hong Kong

## Abstract

*Aim.* To examine the effect of berberine (BBR) on liver fibrosis and its possible mechanisms through direct effects on hepatic stellate cells (HSC).* Methods.* The antifibrotic effect of BBR was determined in a rat model of bile duct ligation- (BDL-) induced liver fibrosis. Multiple cellular and molecular approaches were introduced to examine the effects of BBR on HSC.* Results.* BBR potently inhibited hepatic fibrosis induced by BDL in rats. It exhibited cytotoxicity to activated HSC at doses nontoxic to hepatocytes. High doses of BBR induced apoptosis of activated HSC, which was mediated by loss of mitochondrial membrane potential and Bcl-2/Bax imbalance. Low doses of BBR suppressed activation of HSC as evidenced by the inhibition of *α*-smooth muscle actin (*α*-SMA) expression and cell motility. BBR did not affect Smad2/3 phosphorylation but significantly activated 5′ AMP-activated protein kinase (AMPK) signalling, which was responsible for the transcriptional inhibition by BBR of profibrogenic factors *α*-SMA and collagen in HSC.* Conclusion.* BBR is a promising agent for treating liver fibrosis through multiple mechanisms, at least partially by directly targeting HSC and by inhibiting the AMPK pathway. Its value as an antifibrotic drug in patients with liver disease deserves further investigation.

## 1. Introduction

Hepatic fibrosis is a common pathology in various progressive chronic liver diseases [1]. Fibrogenesis in liver, which often accompanies disease progression from hepatitis to cancerous transformation [2], is an abnormal process in which the organ develops excessive accumulation of extracellular matrix proteins in response to chronic injury [3]. Activation of hepatic stellate cells (HSC) is critical in the fibrogenic process of the liver and activated HSC are known as the main sources of a pathogenic extracellular matrix proteins in liver fibrosis [4, 5]. It is believed that effective treatment of hepatic fibrosis will be both crucial for the prevention of chronic liver failure and beneficial for the prevention of liver cancerous diseases [6]; however, there is still no standard treatment for liver fibrosis [7].

Berberine (BBR) is a natural alkaloid extracted from Coptidis Rhizoma (Huang Lian in Chinese), an herbal drug commonly used in traditional Chinese medicine for treating patients with inflammatory diseases. The major pharmacological actions of BBR may include antimicrobial [8], anti-inflammatory [9], antioxidative [10], and antitumoral activities [11]. Our previous studies have revealed the therapeutic effects of Coptidis Rhizoma and BBR on hepatocellular carcinoma, by inducing apoptotic and autophagic cell death at high doses [12] and by repressing tumor cell motility at lower doses [13]. These observations suggested that BBR be of potential value for treating liver malignancies [14]. Furthermore, it was also found by our group that extract of Coptidis Rhizoma exerted potent protective effect on acute and chronic liver damage in an experimental animal model of liver injury, significantly improving liver function and tissue structure [15, 16]. We further reported that, BBR, a major active compound extracted from Coptidis Rhizoma also protected animal from acute hepatic damage [17, 18]. BBR is able to reduce sustained and chronic liver injury in various animal models of hepatic damage [19]. We have proposed that BBR as an antifibrotic drug and its mechanisms of action are worth further investigation [20].

In this study, we aimed to investigate the in vitro and in vivo antifibrotic effect of BBR and its potential mechanisms with focus on HSC. The antifibrotic activity of BBR was evaluated in bile duct ligation-induced hepatic fibrosis model in rats. The action of BBR on activated HSC was investigated at both nontoxic and toxic levels. Understanding the action and mechanism of BBR in inhibiting hepatic fibrosis may shed light on the further development of antifibrotic treatment.

## 2. Materials and Methods

### 2.1. Chemicals and Reagents

BBR hydrochloride and Compound C, an AMPK inhibitor, were purchased from Sigma-Aldrich (USA).

### 2.2. Animals

Male SD rats of 220~250 g body weight were purchased from Guangdong Medical Laboratory Animal Centre, Guangzhou, Guangdong Province, China. Animals were housed at 25 ± 2°C, with a 12 h light cycle, starting at 06:00, and were provided free access to standard laboratory chow and water. All experiments were approved by the ethics committee of the University of Hong Kong and complied with international guidelines.

### 2.3. Animal Model

Bile duct ligation (BDL) was applied to rats to induce extrahepatic cholestasis-related liver fibrosis. Briefly, under anaesthesia with ether, rats were subjected to ligation of the common bile duct with 3-0 silk and sectioned between the ligatures. The abdominal midline was then closed with catgut. Rats in a sham control group had their bile duct exposed with neither ligation nor sectioning. All rats were caged at 24°C with 12 h : 12 h light-dark cycle and were provided free access to food and water for 7 days before the study. All operated rats except sham controls were randomised into different groups.

### 2.4. Animal Treatment

Rats in sham and model groups received 10 mL/kg of distilled water per day by oral administration. Rats in BBR treatment group received 120 mg/kg/day BBR dissolved in distilled water orally. All treatment lasted for seven weeks.

### 2.5. Biochemical Analysis

At the end of the experiment, animals were sacrificed by i.p. injection of 200 mg/kg pentobarbitone. Blood was collected and serum was separated by centrifugation at 3000 g for 5 min. Serum AST, ALT, and TBil were quantified by a biochemical autoanalyzer. The tissue hydroxyproline (HyP) level was examined with a HyP detection kit (Jiancheng Bioengineering Institute, Nanjing, China) following the manufacturer's instructions.

### 2.6. Histological Analysis

Livers collected from different groups of rats were rinsed with PBS and fixed in 4% buffered formaldehyde for 24 h. Paraffin-embedded tissues were cut into 5 *μ*m sections and stained with hematoxylin and eosin (H&E). To evaluate chronic liver injury by a semiquantitative scoring, five phases of liver injury were defined as follows: S0: no observable scaring; S1: no extended portal area scaring; S2: fibrotic portal area with intact lobule structure; S3: fibrosis with broken lobule structure and no cirrhosis; and S4: cirrhosis. Fibrotic area within 1.5 mm^2^ of each section was measured. Images were captured under light microscope (Leica Microsystems Digital Imaging, Germany) with CCD camera at the magnification of 10 × 10 (Leica DFC 280, Germany).

### 2.7. Cells and Cell Culture

The human hepatocyte cell line L-02 and human HSC line hHS were obtained from Sun Yat-Sen University (Guangdong, China). The cells were cultured in Dulbecco's Modified Eagle medium with high glucose (4.5 g/L) with supplements of 10% Foetal Bovine Serum (FBS) and 1% penicillin/streptomycin and incubated in a humidified atmosphere containing 5% CO_2_ at 37°C.

### 2.8. Cell Viability Assay

Cell viability assay was performed to examine the cytotoxicity of BBR to HSC cells. Briefly, cells were cultured in 96-well cell culture plate in DMEM supplemented with 10% FBS. Each well contained 10,000 cells for attachment overnight. A series of concentrations of BBR were added to the cells to incubate. Then, 10 *μ*L of 3-(4,5-dimethylthiazol-2-yl)-2,5-diphenyltetrazolium bromide (MTT, 5 mg/mL, Sigma, USA) was added to each well 4 h before the end of treatment and the incubation was continued at 37°C. The medium was then discarded and 100 *μ*L DMSO was added to dissolve the crystals with gentle pipetting. The absorbance was read at 575 nm on a Multiskan MS Microplate Reader (Labsystems, Finland).

### 2.9. Annexin V and Propidium Iodide (PI) Staining

Annexin V and PI double staining was introduced to analyze cell apoptosis and necrosis. In brief, cells were trypsinized, collected, and centrifuged. Cells were stained using the Annexin V and PI double staining kit (Sigma-Aldrich, USA) in binding buffer containing 100 mM HEPES/NaOH, 1.4 mM NaCl, and 25 mM CaCl_2_, pH 7.5. Five *μ*L 50 *μ*g/mL FITC-conjugated Annexin V and 10 *μ*L 100 *μ*g/mL PI were added and samples were incubated in dark at room temperature for 15 min. The cell suspension was then detected by flow cytometer (Epics XL, Beckman Coulter, USA). Results were analyzed with the FlowJo software (USA).

### 2.10. Transwell Migration Assay

hHSC motility upon BBR treatment was examined in Millicell-PCF Cell Culture Insert (24-well, 8.0 *μ*m, Millipore). Briefly, the inserts were standing in 24-well cell culture plate. 5 × 10^4^ cells in 100 *μ*L serum-free medium were added to the insert, while 0.5 mL 10% FBS DMEM containing indicated concentrations of BBR was added to each well of the 24-well plate. This was followed by incubation in 5% CO_2_ at 37°C for 24 hr. The noninvading cells on the upper surface were removed by cotton swabs. The cells that invaded across the transmembrane to the lower surface of the membrane were fixed by ice cool 100% ethanol and stained by 2% crystal violet (Sigma-Aldrich, USA). Photographs of the stained migrated cells (3 random fields per culture) were taken under an inverted microscope at 400× and the mean number of cells of the 3 fields was recorded.

### 2.11. JC-1 Staining

The measurement of mitochondrial membrane potential was conducted with JC-1 staining. Cells were seeded in 35 mm glass-bottom dishes and treated with BBR. Cells were then stained with 10 *μ*g/mL JC-1 (Invitrogen, USA) in dark for 30 min and visualized under a fluorescence microscope. Intense red fluorescence indicates the integrity of mitochondrial membrane, while increased green fluorescence represents loss of mitochondrial membrane potential.

### 2.12. Immunofluorescence

Cells were seeded in 35 mm glass-bottom dishes and treated with BBR. The cells were then fixed with 4% paraformaldehyde in PBS followed by incubation with blocking buffer (5% normal goat serum and 0.03% Triton X-100 in PBS). Then, cells were incubated with antibody against *α*-SMA at 4°C overnight followed by wash. Alexa Fluor-conjugated secondary antibody (Invitrogen, USA) was added and cells were incubated in dark for 60 min. Cell nuclei were stained with 4′,6-diamidino-2-phenylindole (DAPI). The cells were observed under fluorescence microscope (Carl Zeiss, USA) and images were captured with a CCD camera (400× magnification).

### 2.13. Reverse-Transcription Quantitative Polymerase Chain Reaction (RT-qPCR)

Total RNA was extracted with total RNA purification kit (Norgen, Canada). cDNA was generated with first-strand cDNA synthesis kit (Roche, USA) using the collected RNA as template. The quantitative PCR was conducted with SYBR Green I master mix (Roche, USA) on LightCycler 480 (Roche, USA). The primer sequences of target genes were listed in Table 1.

### 2.14. Immunoblotting

Cells were lysed with the Radioimmunoprecipitation Assay (RIPA) Buffer with the cOmplete Cocktail Proteinase Inhibitor (Roche, USA) and phosphatase inhibitor (1 mM Na_3_VO_4_ and 1 mM NaF) on ice for 30 min followed by centrifugation at 14,000 rpm at 4°C for 15 min. Supernatants were transferred and protein concentrations were determined by BSA assay (Bio-Rad, USA). Equal yield of protein was separated on SDS-PAGE and transferred onto a polyvinylidene fluoride membrane (PVDF, Bio-Rad). The membrane was then blocked in buffer containing 5% BSA, Tris (10 mmol/L, pH 7.4), NaCl (150 mmol/L), and Tween 20 (1%) at room temperature for 1 hr with gentle shaking. The membrane was then incubated with primary antibodies at 4°C overnight followed by incubation with appropriate secondary antibody (Abcam, UK) at room temperature for 1 hr. The immunoreactivities were detected using ECL advanced kit (GE Healthcare, UK) and visualized using a chemiluminescence imaging system (Bio-Rad, USA).

### 2.15. Statistical Analysis

Data were expressed as mean ± standard deviation of means (SD) and statistical comparisons were conducted using one-way ANOVA. *p* value lower than 0.05 was considered statistically significant.

## 3. Results

### 3.1. BBR Suppressed BDL-Induced Hepatic Fibrosis in Rats

**Figure 1:**
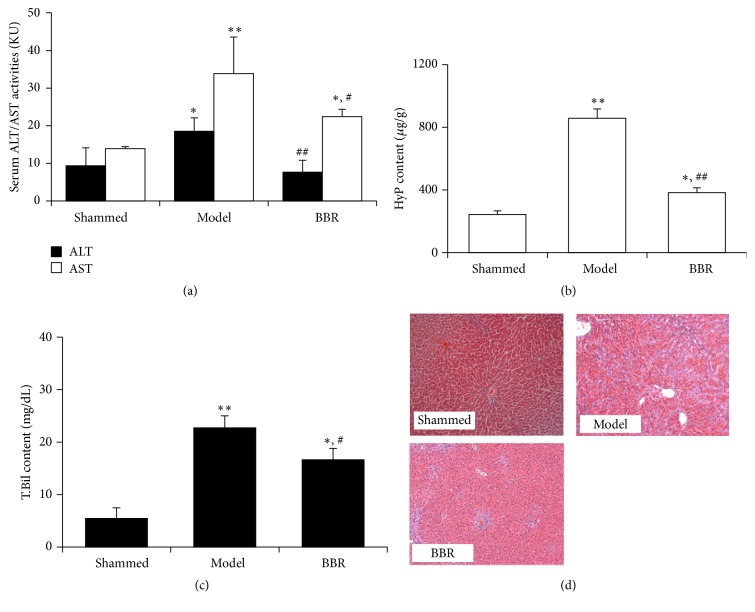
BBR attenuated hepatic fibrosis induced by BDL in rats. The common bile duct of rat was ligated with 3-0 silk and sectioned between the ligatures. Rats in sham-operated control group had their bile duct exposed without ligation or section. Rats were randomised and received either PBS or BBR (120 mg/kg/day orally) for seven weeks. At the end of the study, rats were sacrificed and the serum and liver were collected. (a) BBR reduced serum ALT and AST in BDL rats. (b) BBR reduced the HyP content in the liver. (c) BBR reduced serum total bile acid (T.Bil) in rats with BDL. (d) Histological analysis revealed that the fibrosis was inhibited by BBR treatment. ^*∗*^ *p* < 0.05 and ^*∗∗*^ *p* < 0.01 versus normal group; ^#^ *p* < 0.05 and ^##^ *p* < 0.01 versus the model group.

In the present study, hepatic fibrosis was induced by BDL in rats to induce extrahepatic cholestasis. Significant serum ALT and AST elevation was observed in BDL rats and BBR treatment remarkably reduced serum ALT and AST (Figure 1(a)). Content of HyP in the liver, as a biomarker of hepatic fibrosis, was measured, and BBR potently reduced the liver content of HyP consistently (Figure 1(b)) and reduced serum total bile acid level (Figure 1(c)). Histological analysis revealed that administration of BBR attenuated the hepatic fibrosis induced by BDL in rats (Figure 1(d)). Overall scores of liver fibrosis in different groups are shown in Table 2.

### 3.2. Toxic Doses of BBR Induced Mitochondrial Apoptosis of HSC through Altering Bcl-2/Bax Ratio and Subsequent Caspase Activation

**Figure 2:**
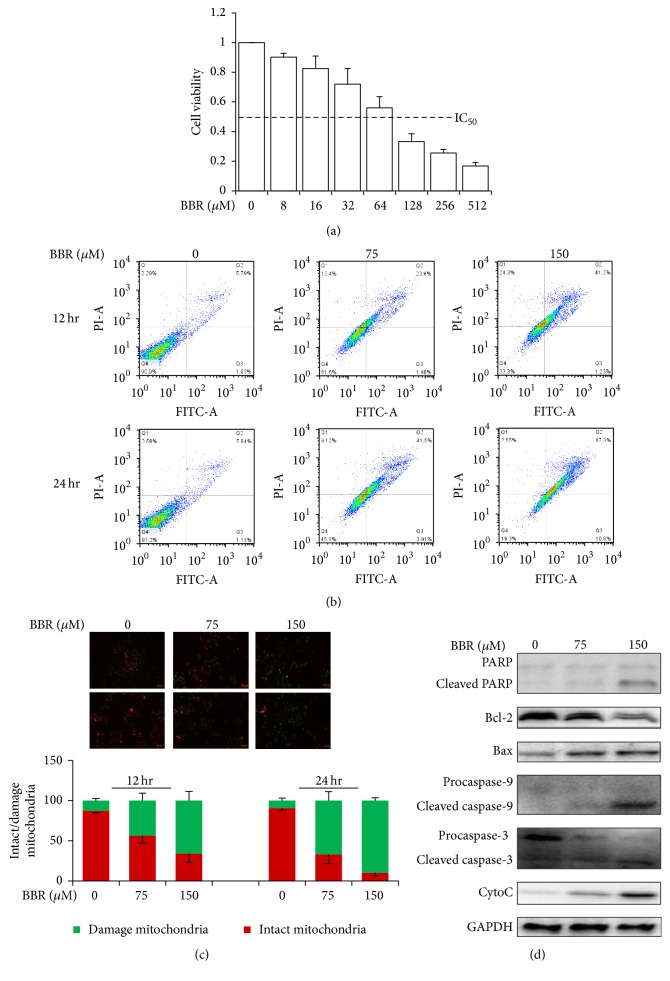
BBR induced apoptosis in constitutively activated hHSC. (a) Constitutively activated hHSC in 96-well plate were treated with BBR for 24 hr and cytotoxicity of BBR was determined by MTT assay. The IC_50_ of BBR on hHSC was roughly 75 *μ*M. (b) hHSC were treated with BBR for 12 hr or 24 hr. Apoptosis and necrosis were analyzed by Annexin V/PI dual staining and flow cytometry. (c) BBR-treated hHSC were stained with JC-1 (10 *μ*g/mL). Significant increase of green fluorescence with loss of red fluorescence indicated the relapse of mitochondrial membrane integrity. (d) BBR regulated Bcl-2/Bax ratio in hHSC. The cells were treated with BBR for 24 hr and cell lysates were subjected to Western blot hybridization. Increase of cleaved caspase-3 and caspase-9 and PARP indicated BBR induced apoptosis, while cyto C released from mitochondria exhibited loss of membrane integrity. Upregulation of Bax with Bcl-2 inhibition was observed in BBR-treated cells.

To explore possible mechanisms of the antifibrotic effect of BBR, we focused on the HSC, which are activated during fibrogenic process. hHSC, a constitutively activated human HSC line, was used to evaluate the cytotoxicity of BBR. High dose of BBR exhibited toxic effects to hHSC (IC_50_ 75 *μ*M, Figure 2(a)). Toxic dose of BBR could initiate apoptosis of hHSC (Figure 2(b)). This effect may be due to the increased permeability of mitochondrial membrane in hHSC with exposure of toxic dose of BBR (Figure 2(c)), which was indicated by the loss of mitochondrial membrane integrity. Expression of Bcl-2 was significantly downregulated upon BBR treatment, leading to Bcl-2/Bax imbalance and mitochondrial membrane polarization (Figure 2(d)). This observation was further confirmed by the release of cytochrome C into cytoplasm, which initiated caspase activation subsequently (Figure 2(d)). These results suggest that toxic dose of BBR may induce mitochondrial apoptosis in hHSC.

### 3.3. Nontoxic Doses of BBR Suppress HSC Activation by Inhibiting *α*-SMA Expression and Cell Migration

**Figure 3:**
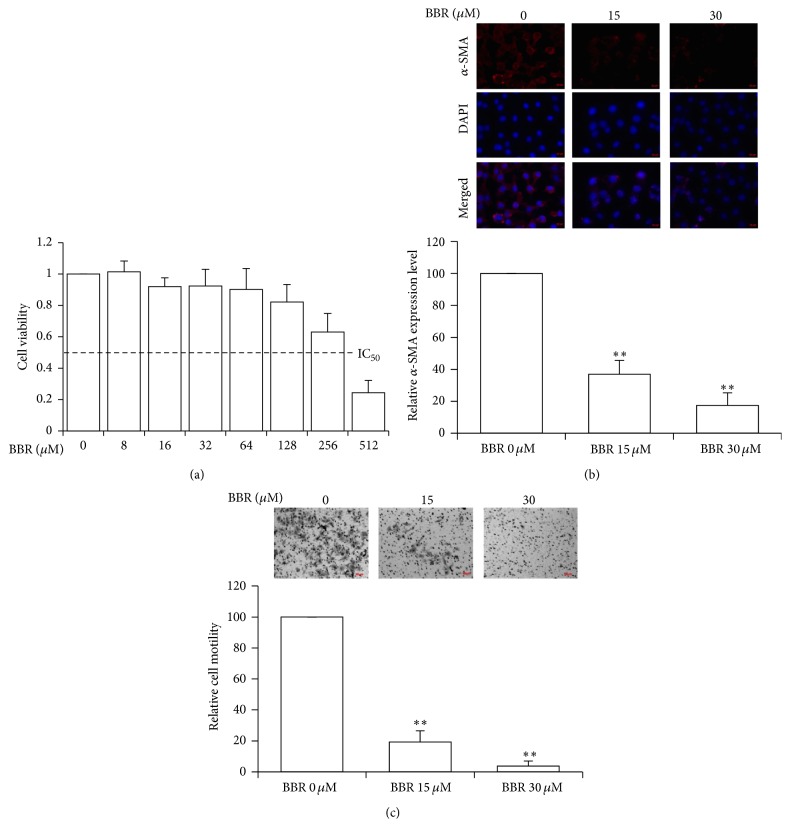
Nontoxic BBR suppressed the activity of hHSC. (a) The normal hepatocyte cell line L-02 in 96-well plates was treated with BBR for 24 hr and cell viability was determined by MTT assay. The IC_50_ of BBR on L-02 cells was around 250 *μ*M. (b) BBR suppressed hHSC migration. Cells in serum-free medium were seeded onto the Transwell and medium containing 10% FBS was used as attractant to initiate cell migration. After different doses of BBR were added, the cells were cultured for 24 hr, fixed with 4% paraformaldehyde, and then stained with 2% crystal violet. Three images were captured for each well and the representative images are shown. BBR treatment significantly reduced cell motility. (c) BBR downregulated *α*-SMA expression in constitutively activated hHSC. Cells were treated with BBR for 24 hr and then fixed with 4% paraformaldehyde. The expression of *α*-SMA was stained (red) and nuclei were stained with Hoechst 33342 (blue). Three images were captured per treatment group and the representative image of each group is shown. BBR treatment significantly reduced *α*-SMA expression in constitutively activated hHSC. ^*∗∗*^ *p* < 0.01 versus control.

The toxic dose of BBR on normal hepatic cell line L-02 was examined to evaluate its possible adverse effect. It was shown that the IC_50_ value of BBR on L-02 cells was about 250 *μ*M, indicating that high doses of BBR may exhibit some toxic action to normal hepatocytes (Figure 3(a)). Further examining the effect of BBR on HSC at nontoxic doses, we found that BBR significantly downregulated the expression of *α*-SMA, the biomarker of HSC activation, indicating that BBR might inactivate the activated hHSC at nontoxic doses (Figure 3(b)). Furthermore, BBR significantly downregulated hHSC motility (Figure 3(c)).

### 3.4. BBR Repressed *α*-SMA and Collagen mRNA Expression without Altering Smad2/3 Phosphorylation

**Figure 4:**
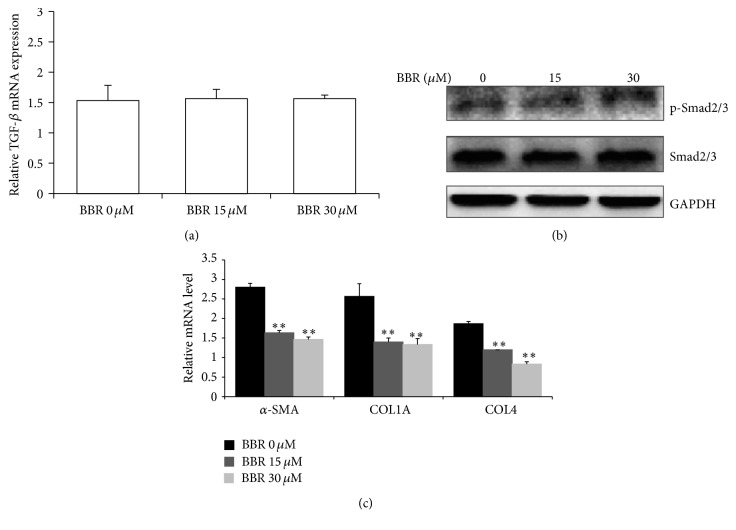
BBR suppressed *α*-SMA, without affecting Smad2/3 phosphorylation. (a) BBR did not affect the TGF-*β* production in hHSC. The expression of TGF-*β* mRNA transcript was analyzed with RT-qPCR. No significant difference between treatment and nontreatment group was observed. (b) BBR did not inhibit Smad2/3 phosphorylation in hHSC. Immunoblotting analysis using specific antibodies revealed no significant difference between treatment and nontreatment group in the phosphorylation of Smad2/3. (c) BBR suppressed the expression of profibrogenic factors. The downstream targets of Smad2/3, fibrogenic *α*-*Sma*,* Col1a1,* and* Col4a3 *were analyzed by RT-qPCR. Significant reduction of *α*-SMA, Col1A1, and Col4a3 mRNAs was observed in BBR-treated cells in a dose-dependent manner. ^*∗∗*^ *p* < 0.01 versus control.

The TGF-*β*/Smad signalling plays an important role in HSC activation in liver fibrosis [21]. As BBR was reported to suppress TGF-*β* expression in the plasma of patients with lung cancer who were undergoing radiotherapy [22], the expression of TGF-*β* was analyzed in our study to prove if similar action of BBR could be found. However, in our study, we observed no significant reduction of TGF-*β* expression in BBR-treated hHSC (Figure 4(a)). Neither did BBR suppress Smad2/3 phosphorylation (Figure 4(b)). However, BBR dose-dependently inhibited the mRNA expression of *α*-SMA (*ACTA2*), collagen 1A (*COL1A1*), and collagen IV (*COL4A3*) (Figure 4(c)).

### 3.5. AMPK Activation Is Responsible for the Smad2/3 Inhibition by BBR in HSC

**Figure 5:**
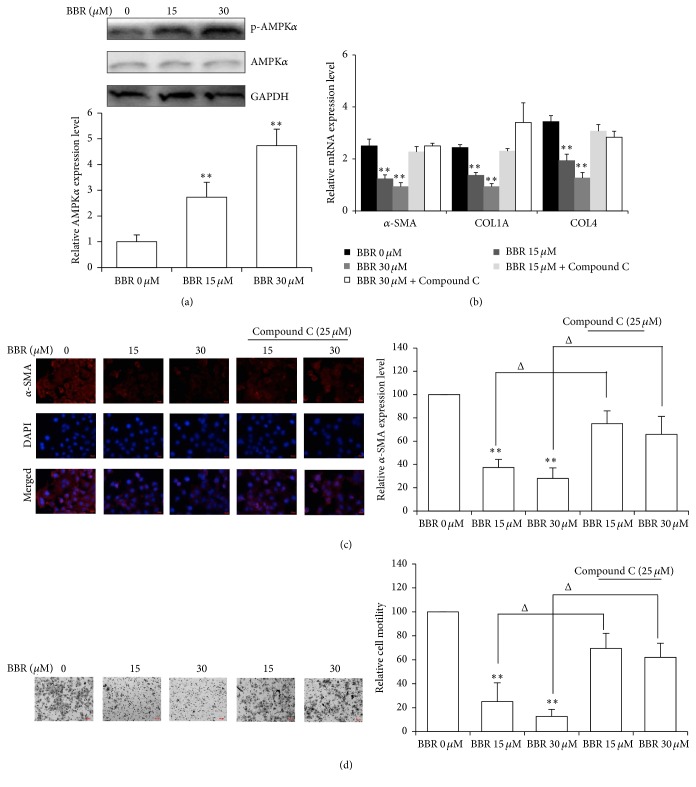
Activation of AMPK was responsible for BBR inhibition of HSC activation. (a) BBR dose-dependently activated AMPK signalling in HSC. (b) Suppression of AMPK with Compound C attenuated inhibition of *α*-SMA, Col1A1, and Col4a3 transcripts by BBR. Cells were treated with BBR alone or in combination with AMPK inhibitor Compound C (25 *μ*M) for 24 hr and then RNA was collected. Expression of *α*-SMA, Col1A1, and Col4a3 mRNA transcripts was analyzed with RT-qPCR. Significant restoration of *α*-SMA, Col1A1, and Col4a3 mRNA expression was found when BBR was given in combination with Compound C. (c) Inhibition of AMPK reactivated BBR-treated hHSC. Significant reduction of *α*-SMA distribution was observed in BBR-treated cells, while recovery of *α*-SMA was found when BBR was given in combination with AMPK inhibitor Compound C (25 *μ*M). (d) hHSC motility was recovered, when cells were treated with BBR in the presence of Compound C (25 *μ*M). ^*∗∗*^ *p* < 0.01 versus control. ^Δ^ *p* < 0.05 versus the same treatment in the absence of Compound C.

Activation of AMPK signalling by BBR was observed in hHSC (Figure 5(a)). Previous studies have exhibited the role of AMPK activation in the treatment against oxidative stress-induced liver injury [23] and it was shown that AMPK activity is critical for the experimental therapy of hepatic fibrosis as well [24]. In our study, further examination was made to explore the role of AMPK activation in BBR's inhibitory effect of experimental hepatic fibrosis. BBR suppression of *α*-SMA, COL1A1, and COL4A3 mRNA in hHSC was potently attenuated by the presence of Compound C, an AMPK inhibitor (Figure 5(b)), suggesting that BBR suppression of these mRNAs might be attributed to AMPK activation in hHSC. A critical role for AMPK in BBR repression of hHSC activation was further confirmed by the fact that BBR repression of *α*-SMA expression and hHSC motility were both prevented by Compound C (Figures 5(c) and 5(d)).

## 4. Discussion

**Figure 6:**
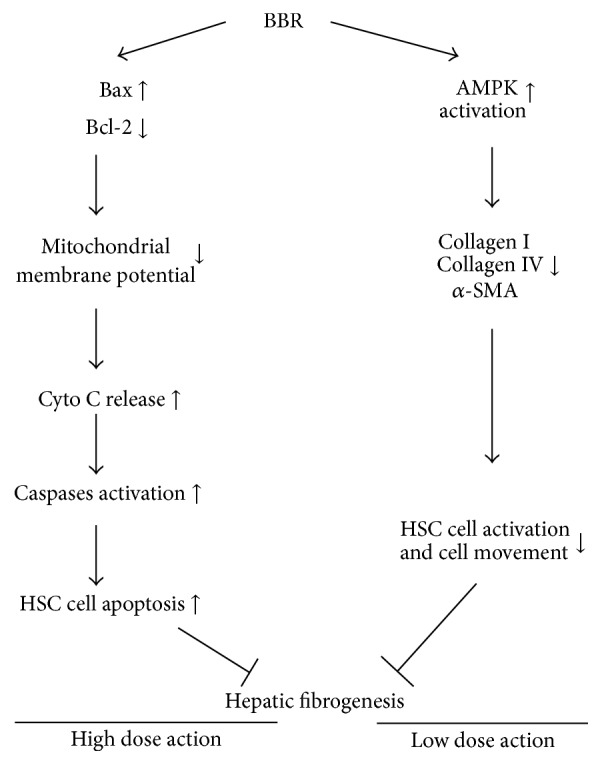
Proposed mechanisms of BBR regulation of HSC.

Critical role of HSC activation in the early development of liver fibrosis has been revealed by previous studies [25]. The activation of HSC initiates its proliferation as well as the production of extracellular matrix (ECM) proteins such as *α*-SMA and collagens [6]. Attempts have been made to explore the use of BBR in the therapy for fibrosis-related hepatic diseases. Previous studies have shown that BBR could be used for the treatment against hypertyraminemia in patients with liver cirrhosis [26], which was correlated with BBR's capacity of reducing blood lipid in hyperlipidemic patients [27]. Experimental studies have been also conducted, the results of which exhibit the potential of BBR in ameliorating hepatic fibrosis with various mechanisms [28, 29]. In particular, it was shown that the antioxidative activity of BBR contributes to improvement of experimental hepatic fibrosis via stimulating matrix metalloproteinase-2 (MMP-2) [30]. Our findings further showed that apoptosis of HSC could be initiated by toxic doses of BBR, and Bcl-2/Bax-mediated mitochondrial membrane potential loss may be involved in induction of HSC apoptosis by BBR. Interestingly, we found that nontoxic doses of BBR downregulated the activation of HSC, suppressing production of ECM proteins, preventing deregulation of hepatic architecture and protecting hepatocytes from hepatocellular dysfunction (Figure 6) [31].

It has been previously shown that the activity of AMPK signalling is a critical factor in the prevention of hepatic fibrogenesis. In AMPK-deficient mice, the fibrogenesis and liver fibrosis could be enhanced dramatically, and initiation of AMPK could suppress HSC proliferation and collagen expression [31]. The preventive effect of AMPK on hepatic fibrosis was further evidenced by the fact that an adipocytokine adiponectin could disrupt leptin-mediated hepatic fibrosis through the activation of AMPK in HSC [24]. A recent study showed that berberine can replenish the activity of AMPK in the liver of carbon tetrachloride- (CCl_4_) treated mice [32]; however, whether activation of AMPK by berberine is responsible for the improvement of experimental fibrosis remains not clear. Furthermore, whether activation of AMPK by berberine can suppress activated hepatic stellate cells, which majorly mediates fibrogenesis in the liver, was not studied. In our study, we observed that hepatic fibrosis induced by BDL could be attenuated by BBR, which in hHSC activates AMPK signalling and inhibits hHSC migration and *α*-SMA production. Inhibition of AMPK by Compound C potently reduced the inhibitory effect on hHSC activation, which was evidenced by the fact that cell motility and *α*-SMA expression were restored in BBR-treated cells in the presence of Compound C. These findings support a central role of AMPK activation in BBR's effect on HSC activation and subsequent fibrogenesis.

We noticed that BBR has no effect on the signal transduction of TGF-*β*/Smad. BBR neither suppresses the TGF-*β* expression in HSC nor inhibits Smad2/3 phosphorylation. However, the expression of profibrogenic factors *α*-SMA, COL1A1, and COL4A3 was inhibited by BBR and this effect could be attenuated by inhibition of AMPK by Compound C. These observations indicate that activation of AMPK could repress fibrogenesis without affecting Smad2/3 phosphorylation. A previous study showed that transcription activity mediated by Smad2/3 requires cooperation of transcription coactivators CBP and p300, which initiate the N-terminal acetylation of Smad2/3 [33, 34]. Phosphorylated AMPK was reported to bind with p300, initiate its proteasomal degradation, and consequently inhibit Smad2/3 transcription activity without affecting their phosphorylation [35]. Deletion of p300 has been found to suppress fibrogenic collagen type I and *α*-SMA expression and lead to fibrogenesis inhibition [35, 36]. Our findings on the critical role for BBR activation of AMPK in HSC shed light on the antifibrotic action of BBR and support its potential value for the treatment of fibrosis in liver disease.

## 5. Conclusion

BBR is a promising agent for treating liver fibrosis through multiple mechanisms, at least partially by directly targeting HSC and by inhibiting the AMPK pathway. Its value as an antifibrotic drug in patients with liver disease deserves further investigation.

## Figures and Tables

**Table 1:** Primer sequence.

	Forward	Reverse
*TGFB1*	TGAACCGGCCTTTCCTGCTTCTCATG	GCGGAAGTCAATGTACAGCTGCCGC
*ACTA2*	CCGACCGAATGCAGAAGGA	ACAGAGTATTTGCGCTCCGAA
*COL1A1*	CAGCCGCTTCACCTACAGC	TTTTGTATTCAATCACTGTCTTGCC
*COL4A3*	GCTGTCAACACCAGCTCTGA	CGGTGCACCTGCTAATGTAA
*ACTB*	CCAACCGCCAGAAGATGA	CCAGAGGCGTACAGGGATAG

**Table 2:** Histological analysis of the effect of BBR on BDL-induced liver fibrosis in rats.

Group	Sample (*n*)	Fibrosis stage					Fibrotic area within 1.5 mm^2^
		S0	S1	S2	S3	S4	
Shammed	6	6	0	0	0	0	0.007 ± 0.005
Model	6	0	1	1	3	1	0.079 ± 0.025^**∗∗**^
BBR (120 mg/kg)	6	0	3	2	1	0	0.032 ± 0.024^*∗*,#^

*∗*: *p* < 0.05, *∗∗*: *p* < 0.01 versus shammed group; #: *p* < 0.05 versus model group.
